# Electrokinetic Manipulation of Biological Cells towards Biotechnology Applications

**DOI:** 10.3390/mi15030341

**Published:** 2024-02-29

**Authors:** Songyuan Yan, Zarya Rajestari, Timothy Clifford Morse, Harbour Li, Lawrence Kulinsky

**Affiliations:** 1 Materials and Manufacturing Technology, University of California Irvine, 5200 Engineering Hall, Irvine, CA 92627, USA; songyuy1@uci.edu; 2 Mechanical and Aerospace Engineering, University of California Irvine, 5200 Engineering Hall, Irvine, CA 92627, USA; zrajesta@uci.edu (Z.R.); tcmorse@uci.edu (T.C.M.); zihehl@uci.edu (H.L.)

**Keywords:** dielectrophoresis, electrokinetic guidance, microfluidic device, single cell analysis

## Abstract

The presented study demonstrates the capability of the template-based electrokinetic assembly (TEA) and guidance to manipulate and capture individual biological cells within a microfluidic platform. Specifically, dielectrophoretic (DEP) focusing of K-562 cells towards lithographically-defined “wells” on the microelectrodes and positioning singles cells withing these “wells” was demonstrated. K-562 lymphoblast cells, are widely used in immunology research. The DEP guidance, particularly involving positive DEP (pDEP), enables the controlled guidance and positioning of conductive and dielectric particles, including biological cells, opening new directions for the accurate and efficient microassembly of biological entities, which is crucial for single cell analysis and other applications in biotechnology. The investigation explores the use of glassy carbon and gold as electrode materials. It was established previously that undiluted physiological buffer is unsuitable for inducing positive DEP (pDEP); therefore, the change of media into a lower ionic concentration is necessary. After pDEP was observed, the cells are resubmerged in the Iscove’s modified Dulbecco’s medium (IMEM), a cell culturing media, and incubated. A dead/alive staining assay was performed on the cells to determine their survival in the diluted buffer for the period required to capture them. The staining assay confirmed the cells’ survival after being immersed in the diluted biological buffer necessary for electrokinetic handling. The results indicate the promise of the proposed electrokinetic bio-sorting technology for applications in tissue engineering, lab-on-a-chip devices, and organ-on-a-chip models, as well as contributing to the advancement of single cell analysis.

## 1. Introduction

Microassembly and, specifically, electrokinetic handling have emerged as critical enabling techniques in biology, allowing for the manipulation and assembly of microscopic components at the microscale with precision [1,2]. One important application of electrokinetic microassembly in biology is the assembly and sorting of single cells, which allows researchers to create well-defined cellular arrangements for tissue engineering and drug screening [3,4]. Moreover, microassembly-based techniques have facilitated the development of efficient biosensors for single-cell analysis, enabling the real-time monitoring of cellular activities [4].

**Figure 1:**
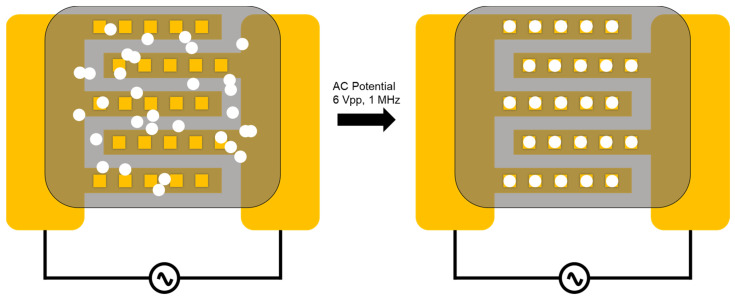
The schematic of DEP capturing of microparticles. The gold electrodes are presented in yellow. Before the AC potential is applied, the microparticles (represented in white) are distributed throughout the liquid volume (**left**). Once the bias with an appropriate AC frequency is applied, the particles are trapped within windows on the electrodes under the influence of the DEP force (**right**). The gray area represents a layer of photoresist which blocks the electric field.

Furthermore, the parallel assembly approach utilizing electrokinetic guidance has been demonstrated as a powerful strategy for the manipulation of microparticles [5]. Microfluidic electrokinetic systems, which exploit the electrical properties of fluids and particles for accurate control of movement and positioning, have found numerous applications in lab-on-a-chip devices and biomedical research [6]. By utilizing different types of electrokinetic effects, such as electroosmosis (EO), dielectrophoresis (DEP), and electrophoresis (EP), researchers can precisely control the motion and alignment of components such as biological cells or dielectric microbeads within microfluidic systems [5] (see the schematics of electrokinetic assembly in Figure 1). DEP handling and guidance has increasingly been employed in the field of bio- and microassembly, due to its ability to manipulate dielectric particles or particles with a zero net charge [7,8]. Dielectrophoretic force causes the movement of polarizable particles, such as cells or biomolecules, in a non-uniform electric field. The DEP allows for non-contact and, thus, the massively parallel simultaneous manipulation of many thousands of biological particles separated by their size, shape, and dielectric properties. The selection of the subgroup of particles for manipulation can be achieved by varying the frequency of the applied AC bias. This approach enables the selective separation and concentration of cells, organelles, or biomolecules within complex biological samples, an ability which is essential for various applications, including diagnostics, cell therapy, and drug discovery [9].

The integration of electrokinetic approaches, DEP or EO, with microwell arrays for single-cell segregation marks a significant advancement over traditional sequential deposition methods. Traditional techniques, often mechanical or hydrodynamic, handle cells one by one, limiting operating efficiency and potentially causing cellular damage due to direct contact [10]. In contrast, the electrokinetic manipulation of cells towards individual microwells employs a parallel assembly strategy using an external electric field to simultaneously manipulate thousands of cells. This method enhances efficiency and minimizes mechanical stress on cells, crucial for applications requiring preserved cell viability. Also, traditional microfluidic strategies for single-cell segregation rely heavily on hydrodynamic properties, utilizing fluid flow to isolate and manipulate cells. Managing the fluid dynamics in hydrodynamic systems can be complex, requiring precise control of flow rates and pressures. This approach complicates the setup and makes the system more challenging to use and less accessible for some applications [11,12,13,14]. In contrast, the microwell approach primarily leverages electrode geometry to exert DEP and EO forces, enabling precise control over cell positioning without the need for complex fluid dynamics. This method offers a unique advantage: the physical structure of the microwells, along with the applied DEP force, ensures that cells are reliably trapped within each well. The walls of the microwells act as physical barriers, making it difficult for the cells to move out unintentionally. This approach simplifies the process of single-cell segregation and makes it possible to rapidly segregate and trap multiple cells within individual wells/microreactors for further application of single cell analysis, drug testing, genetic manipulation, etc.

In this work, a strategy leveraging DEP for trapping single cells within microscopic wells is demonstrated. By utilizing DEP, cells can be selectively trapped and positioned based on their dielectric properties, size, and shape. Furthermore, the DEP approach can aid in the development of organ-on-a-chip models by allowing the assembly of multiple cell types in spatially organized microenvironments, mimicking the native tissue architecture [15]. Overall, the DEP-based trapping of single cells in microscopic wells has the potential to improve various aspects of biological research and contribute to the advancement of precision medicine and regenerative therapies.

## 2. Materials and Methods

### 2.1. Glassy Carbon IDEAs

Glassy carbon interdigitated electrode arrays (IDEAs) and gold IDEAs were used in the experiments. The carbon IDEA fabrication was performed on 4 in. diameter silicon wafers covered with a deposited 1 μm thick layer of thermal oxide (University Wafer, Boston, MA, USA). A SU-8 2025 photoresist (Kayaku Advanced Materials, Inc., Westborough, MA, USA) layer was spin-coated onto the oxide layer using a Laurell photoresist spinner (Laurell Technologies, Lansdale, PA, USA). The initial angular velocity of the spinner was 500 rpm for 10 s, and then it was increased to 4000 rpm for another 30 s. Next, the sample was soft-baked at 95 °C for 5 min using a Dataplate Pmc 732 hot plate (American Laboratory Trading, East Lyme, CT, USA). The IDEAs were then patterned onto the photoresist using a UV flood exposure system (Oriel Instrument, Newport Corp., Stratford, CT, USA). The sample was exposed to a 2 mW/cm^2^ intensity of UV light for 80 s under a patterned photomask (CadArt, Bandon, OR, USA). The sample was post-baked at 65 °C for 1 min and then 95 °C for 5 min on the hot plate. The photoresist layer was developed using an SU-8 developer (Kayaku Advanced Materials, Inc., Westborough, MA, USA). The sample was then hard-baked at 95 °C for 30 min. The remaining photoresist was pyrolyzed into the glassy carbon electrode arrays using a pyrolysis furnace (Thermo Fisher Scientific, Thermo Scientific, Waltham, MA, USA) at 900 °C for 60 min. The pyrolyzed glassy carbon IDEAs were characterized using a Dektak 3 profilometer (Veeco Instrument Inc., Fullerton, CA, USA). The interdigitated carbon fingers’ height was measured to be between 1.5–2.0 μm and the width was measured to be around 100 μm. The gap between fingers was 100 μm [5].

### 2.2. Gold IDEAs

Gold IDEAs were also fabricated on top of 4 in. silicon wafers. First, a Shipley 1827 photoresist (Shipley Company LLC, Tustin, CA, USA) layer was spin-coated onto the surface at 3000 rpm for 30 s using a Laurell photoresist spinner. Next, the sample was soft-baked at 95 °C for 30 min. Next, lithography was performed by exposing the wafers to a 10 mW/cm^2^ UV light for 35 s using a Karl Suss MA6 Mid/Deep UV Mask Aligner (SUSS MicroTec, Garching bei München, Germany). The sample was exposed under the same photomask used for carbon microelectrodes. The wafers were then developed with MF-319 developer (Shipley Company LLC, Tustin, CA, USA). Next, a 500 Å Chromium (Cr) adhesion layer from 99.95% chromium granules (Kurt J. Lesker Company, Jefferson Hills, PA, USA) was deposited onto the wafer using a CHA-600S/CV-8 thermal e-beam evaporator (Ferrotec temescal systems, Livermore, CA, USA), followed by the deposition of a 3000 Å Gold (Au) layer from 99.99% gold pellets (Kurt J. Lesker Company, Jefferson Hills, PA, USA) using the same e-beam deposition technique. The final metal pattern was left on the structure after a lift-off process by submerging the wafer in an acetone solution in a Branson CPX2800H Ultrasonic Digital Bench (Emerson Electric, St. Louis, MO, USA) [5].

### 2.3. Creating Windows in the Resist Layer on IDEAs

**Figure 2:**
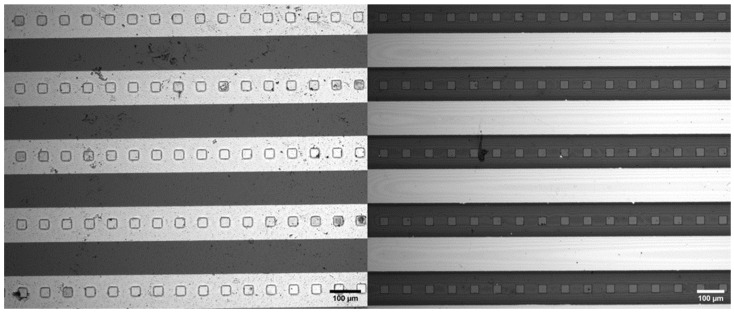
The electrodes were observed under Nikon Eclipse microscope; the gold interdigitated electrode array (**left**) and the carbon IDEA (**right**). All the electrodes are 120 microns wide with 120-micron gaps and windows in the resist that are 30 μm wide squares.

A layer of SU-8 photoresist was coated over the IDEAs and lithographically patterned to form an array of windows in the photoresist layer. Figure 2 presents the optical micrograph of the IDEAs where each 120 μm wide electrode finger is separated by a 120 μm gap between the adjacent fingers. The design of the patterned photoresist layer consists of an array of square windows of 30 μm. These wells were produced via conventional lithography techniques utilizing an iron oxide mask (Front Range Photomask, Las Vegas, NV, USA) and a MA 6 mask aligner (SUSS MicroTec Inc., Corona, CA, USA). The thin layer of SU-8 2002 photoresist was spin-coated on the surface of the IDEA, and the subsequent soft-bake, exposure, and postexposure bake processes were all adjusted depending on the desired height of the resist layer. A photoresist layer 6 μm high was soft-baked for 2 min at 95 °C, exposed for 4 s at an energy intensity of 10 mW/cm^2^ using the MA6 mask aligner’s light source, and post-baked for 3 min. The wafer was then hard-baked at 95 °C for 45 min. The second layer was then post-baked for 5 min at 95 °C. Afterward, the un-cross-linked regions of the photoresist were etched away using the same SU-8 developer for 5 min. Lastly, the second layer was hard-baked at 95 °C for 45 min [5]. The final structures of the micro-windows are displayed in Figure 2.

### 2.4. Cells Processing

K-562 suspension cells were cultured using Iscove’s modified Dulbecco’s medium (IMEM) (Gibco™, ThermoFisher, Waltham, MA, USA) with 10% fetal bovine serum (ThermoFisher, Waltham, MA, USA) and 1% penicillin-streptomycin (ThermoFisher, Waltham, MA, USA) and incubated at 37 °C in an incubator with 5% CO_2_ and appropriate humidity. After the cells reached confluency, they were centrifuged at a speed of 1000 rpm for 5 min. Following the removal of the media, the cells were suspended in 5 mL of fresh media and counted using a hemocytometer. The required volume of the cell suspension in the culturing media (IMEM) was centrifuged and subsequently suspended in various media. These media included: deionized (DI) water; DEP Medium (8.50% (*w*/*v*) consisting of 0.248 mM sucrose (0.248 mOsm/L), 0.50% (*w*/*v*), 27.8 mM dextrose (27.8 mOsm/L), 100 μM CaCl_2_ (300 μOsm/L), and 250 μM MgCl_2_ (750 μOsm/L) dissolved in DI water, adjusted to a conductivity of 0.01 S/m using PBS buffer with overall osmolarity of approximately 30.0 mOsm/L) [16]; phosphate-buffered saline (PBS) buffer (300 mOsm/L) (Gibco™, ThermoFisher, Waltham, MA, USA); Iscove’s modified Dulbecco’s medium (IMEM) with 10% fetal bovine serum and 1.00% penicillin-streptomycin (Gibco™, ThermoFisher, Waltham, MA, USA); Dulbecco’s modified Eagle medium (DMEM) (Gibco™, ThermoFisher, Waltham, MA, USA). These media were selected because they are common solutions used in cell passage and cell analysis to provide a stable pH environment and to maintain cells’ viability.

### 2.5. Experimental Setup

**Figure 3:**
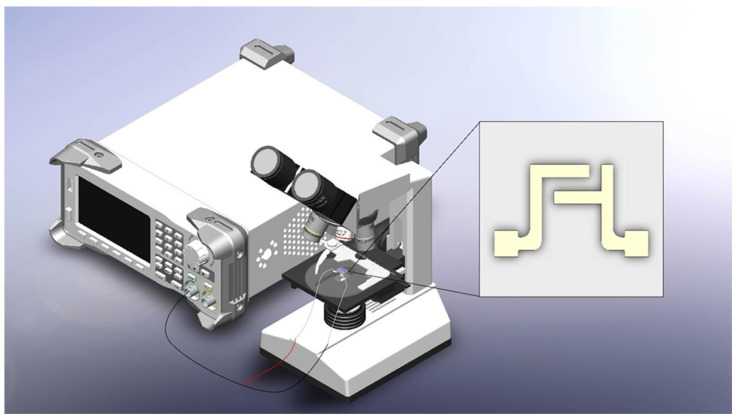
The physical system set-up for the microfluidic electrokinetic experiments. The microscope is also connected to a sCOMS camera to convert optical data into digital data. The inset represents the schematic of IDEA covered with the layer of resist with the windows opened in the resist layer.

Both the carbon IDEAs and the gold IDEAs were soldered at contact pad with buss wires (All electronics, Van Nuys, CA, USA) using Indium solder. The buss wires were then connected to a function generator (Stanford Research System, Sunnyvale, CA, USA) (see Figure 3). A volume of 4 μL of the prepared cell solution (1 million cells in 1 mL media) was pipetted to the top of the electrode within a mini petri dish. The observation and recording began once the function generator was set to generate the desired AC frequencies, with a peak-to-peak voltage of 6 or 20 Vpp and an offset voltage of 0 V. The observations were conducted with a Nikon Eclipse microscope (Nikon, Minato, Japan) and a SPOT RT sCMOS camera (Diagnostic Instruments, Inc., Sterling Heights, MI, USA). The SPOT Basic video editing program (SPOT Imaging, Sterling Heights, MI, USA) was used to record the observations. The recording was collected by recording the computer screen using Camtasia 2018 Recorder software (TechSmith Software Company, East Lansing, MI, USA).

### 2.6. Viability Test

A viability test was conducted on a Neubauer improved hemocytometer. A volume of 10 µL was taken from the cell concentration, and it was mixed with 10 µL of Trypan blue stain (0.4%) (Gibco™, ThermoFisher, Waltham, MA, USA). A volume of 10 µL of the mixture was placed on the hemocytometer to perform the cell containing following the hemocytometer instructions. The viability test was performed in five groups: 1 million K-562 cells in 1 mL DEP medium, 1 million K-562 cells in 1 mL IMEM medium, 1 million K-562 cells in 1 mL DEP medium and applied 20 Vpp 1 MHz AC potential for 5 min, 1 million K-562 cells in 1 mL IMEM medium and applied 20 Vpp 1 MHz AC potential for 5 min. The last testing group is 1 million K-562 cells in 1 mL DEP medium and applied 20 Vpp 1 MHz AC potential for 5 min, immediately introduced with 3 mL of IMEM. The 4 mL mixture was centrifuged for 5 min at 1000 rpm. The medium was replenished with 1 mL fresh IMEM medium. All samples were incubated at 37 °C in an incubator with 5% CO_2_ and appropriate humidity. The viability tests were conducted 1 h and then 1 day after the incubation.

### 2.7. Dielectrophoresis

DEP is a phenomenon that arises from the interaction of a non-uniform electric field with the induced dipole moment of polarizable particles suspended within a medium. The physics of DEP is based on the disparity between the permittivities and conductivities of the particles and the surrounding medium. When a non-uniform electric field is applied, the particles experience a force that either attracts or repels them from the regions of high electric field strength, depending on their polarizability relative to the medium.

**Figure 4:**
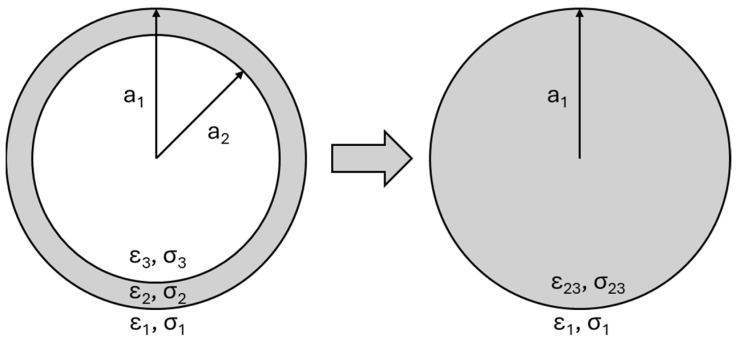
Schematic of the cross-sectional view of a biological cell with a single shell, featuring two distinct internal permittivities and conductivities. Through the shell model, this complex internal structure is simplified and viewed as a uniform sphere. This approach yields new, frequency-dependent values for both permittivity and conductivity [17].

The governing equation of the DEP force experienced by a polarizable particle in a non-uniform electric field is represented by Equation (1) [17]:

F_DEP_ = 2πr^3^ε_m_ Re {CM(ω)} ∇|E|^2^ (1)

(2) $$\mathrm{CM}(\omega )=\{({\tilde{\varepsilon }}_{23}(\omega )-{\tilde{\varepsilon }}_{m}(\omega ))/({\tilde{\varepsilon }}_{23}(\omega )+2{\tilde{\varepsilon }}_{m}\cdot (\omega ))\}$$

(3) $${\tilde{\varepsilon }}_{23}={\tilde{\varepsilon }}_{2}\left[\gamma_{12}^{3}+2\left(\frac{{\tilde{\varepsilon }}_{3}-{\tilde{\varepsilon }}_{2}}{{\tilde{\varepsilon }}_{3}+2{\tilde{\varepsilon }}_{2}}\right)\right]/\left[\gamma_{12}^{3}-\left(\frac{{\tilde{\varepsilon }}_{3}-{\tilde{\varepsilon }}_{2}}{{\tilde{\varepsilon }}_{3}+2{\tilde{\varepsilon }}_{2}}\right)\right]$$

(4) $$\tilde{\varepsilon }\cdot (\omega )=\varepsilon -i\sigma /\omega$$

(5) $$\gamma_{12}=a_{1}/a_{2}$$

where r is the particle radius; ε_m_ and ε_23_ are the permittivity of the medium and the cell, respectively; E represents the magnitude of the electric field; ω is the angular frequency of the electric field; and Re {CM(ω)} is the real part of the complex Clausius–Mossotti (CM) factor, which describes the relative polarizability of the particle and the medium and is dependent on the complex permittivity and electric conductivity. The complex permittivities, $\tilde{\varepsilon }$, of both the cell and the medium are as a function of the real permittivies and electrical conductivity of the cells or the medium and also on the variable applied AC frequency. As a result, a positive Re {CM(ω)} indicates that the particle is more polarizable than the medium, resulting in pDEP, which attracts the particle toward regions of high electric field strength. Conversely, a negative Re {CM(ω)} signifies that the particle is less polarizable than the medium, leading to negative DEP (nDEP), which repels the particle away from the high electric field region. Equation (3) is the equation that represents the multiple shells model in a homogeneous spherical particle of radius a_1_ and permittivity ${\tilde{\varepsilon }}_{23}.$ The integration model is demonstrated in Figure 4 [17,18].

## 3. Results

**Figure 5:**
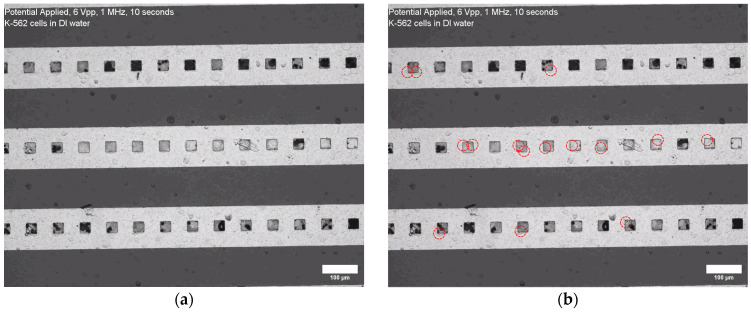
The results of the electrokinetic capturing of K-562 cells within 30-micron wells on the gold IDEAs. Image (**a**) presents the volume of cells before application of the electrical bias. Image (**b**) presents the movement of the cells (circled in red) towards the wells when the AC signal of 1 MHz and 6 Vpp was applied for 5 s. The experiment was conducted in DI water. Recorded video is shown in the Supplementary Materials.

Figure 5 demonstrates the results of the electrokinetic capture of individual K-562 cancer cells within the 30-micron windows (or “wells”) opened in the layer of the photoresist above the electrodes. The cells were captured utilizing pDEP under the bias of 1 MHz and 6 Vpp of the AC signal applied for 5 s. The strength of the DEP was not sufficient to quickly attract the cells from the bulk solution into the wells, and the cells that precipitated on the bottom of the microfluidic volume at the level of the electrodes were quickly (within 5 s) attracted into the wells, while it took much longer to attract the suspended cells.

## 4. Discussion

### 4.1. Simulation

**Figure 6:**
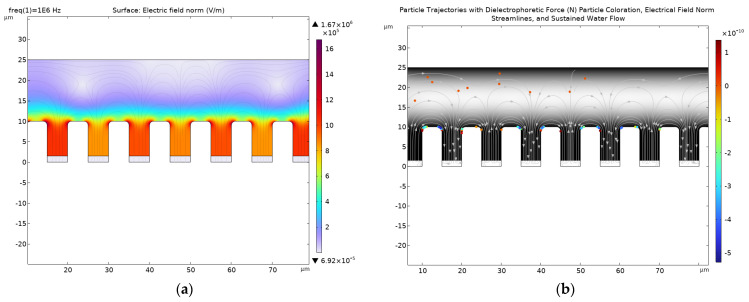
The simulation results of the DEP microfluidic system via COMSOL Multiphysics 6.2. (**a**) The electric field simulation of the electrokinetic microfluidic system; (**b**) the particle tracing simulations of the microfluidic system. The microparticles are attracted to the edges of the micro-windows where the electric field is the strongest.

The microfluidic system was simulated via COMSOL Multiphysics 6.2 utilizing the electric current module, creeping flow module, and particle tracing of fluid flow module. The geometry represented a cross-section view of the experimental system. The windows are displayed as trenches in the simulation. The simulations are presented in Figure 6. In Figure 6a, an electric field simulation result is presented. The color bar represents the intensity of the electric field. As indicated, the electric field is strongest at the bottom of the window. In Figure 6b, the particle tracing result demonstrated how the cells or microparticles were captured inside the micro-windows via pDEP.

### 4.2. Experimental Findings

The experiments demonstrated that the biological cells K-562 in PBS buffer (with the conductivity of 13.5–17.0 mS/cm), IMEM (with the conductivity of 1–2 mS/cm), and DMEM (with the conductivity of 1.68 S/m) experience only negative DEP (nDEP) [18,19]. However, when the ionic concentration of the buffers decreases, pDEP of the biological cells can be achieved. If cells are placed in deionized water for a prolonged period of time, the swelling and bursting of cells will follow as a result of osmosis. The cells were centrifuges in their original buffer solution and when the cells precipitated to the bottom of the beaker, all solution was removed and replaced with DI water or DEP medium. It was observed that pDEP only occurs when 1.0 mL or more DI water was added to reduce the ionic concentration. The suspension of cells was transferred to a sterile mini petri dish containing the IDEA chip connected to the function generator. In order to facilitate the ability to predictably move particles and cells, an external electric field was applied to the IDEAs. Within the DEP medium, the K-562 cells experience pDEP at 1 MHz and EO at 10 kHz. EO is observed with the clear pattern of the cells in a circulating flow emanating around the edges of the electrode, as observed by other authors [20,21]. Another characteristics behavior of EO is that unlike pDEP, which attracts the particles towards the edge of the electrode, the EO vertex drags the particles past the edge of electrode, and due to inertia, particles gather at the center of the electrode [5], also seen in the present study at 10 kHz.

The viability test results are presented in Table 1. The viable cells population in DEP solution is reduced from 1 million cells to 240,000 cells after 1 h. If cells, in addition to being immersed in DEP solution, are exposed to the potential of 20 Vpp applied for 5 min, the viable cell population is reduced from 1 million cells to 120,000 cells after 1 h. Electrokinetic manipulation of microparticles and biological cells can be achieved in less than a minute under the bias of 4 to 10 V (for example, as Figure 6 indicates, a potential of 6 V is sufficient to achieve electrokinetic cell manipulation and sorting), so the application of 20 Vpp for 5 min represents a conservative testing condition. Additionally, it is likely that some cell damage is sustained due to centrifugation where the cells are sedimented before the media is changed to IMEM (see Column 5 of Table 1). The viability test at 1 day indicates cell growth, demonstrating the success of the media replacing strategy. It is evident from the viability test results of Table 1 that the immersion of the cells in a low-ionic-concentration solution such as DEP medium is detrimental to cells’ health, while cells thrive in IMEM solution. Therefore, the automation of solution exchange from DEP medium to IMEM immediately after electrokinetic manipulation will be the focus of subsequent studies.

For gold electrodes, the strongest pDEP occurs at 1 MHz, while for glass carbon electrodes, the strongest pDEP occurs at approximately 800 kHz. The pDEP frequency shift related to electrode material differences is due to polarization impedance. Glass carbon, with finite electric permittivity, requires a longer relaxation time and lower switching frequencies to reach maximum polarization [17].

## 5. Conclusions

It was demonstrated that DEP electrokinetic handling is a promising technique for capturing and manipulating individual cells in microfluidic systems. The successful manipulation of K-562 cells and their survival after DI water or DEP medium dilution and electrokinetic processes further support the potential of this method in various biological applications. A simulation of the microfluidic system represented microparticle behavior under the influence of electrokinetic manipulation. These findings have significant implications for tissue engineering, lab-on-a-chip devices, and organ-on-a-chip models, as well as contributing to the advancement of precision medicine and regenerative therapies. Future research will focus on optimizing the DEP parameters and design of the microfluidic system to enhance the efficiency of the electrokinetic handling of biological cells for several applications such as single cell analysis and tissue engineering.

## Figures and Tables

**Table 1:** Viability test results. All five samples have an initial concentration of 1 million K-562 cells in the corresponding media. The viability tests were conducted 1 h and 1 day after the cell culturing. The third and fourth columns reflect the results of experiments where a voltage of 20 Vpp, 1 MHz of AC potential was applied for 5 min to achieve the dielectrophoretic guidance of cells. The fifth column reflects the viability of cells that were exposed for 5 min to 20 Vpp bias in DEP medium, and then the cells were moved to IMEM solution. The viability tests were conducted with Trypan blue stain and cell counting was performed with a hemocytometer.

DEP Medium	IMEM Medium	DEP Medium 20 Vpp	IMEM Medium 20 Vpp	DEP Medium 20 Vpp Followed by Replacement of DEP Medium by IMEM
1 h	1 h	1 h	1 h	1 h
240,000	1,100,000	120,000	1,320,000	80,000
1 d	1 d	1 d	1 d	1 d
4000	1,460,000	8000	740,000	140,000

## Data Availability

The data presented in this study are available on request from the corresponding author.

## Supplementary Materials

The following supporting information can be downloaded at: https://www.mdpi.com/article/10.3390/mi15030341/s1.
